# Near-Infrared Blood Vessel Image Segmentation Using Background Subtraction and Improved Mathematical Morphology

**DOI:** 10.3390/bioengineering10060726

**Published:** 2023-06-15

**Authors:** Ling Li, Haoting Liu, Qing Li, Zhen Tian, Yajie Li, Wenjia Geng, Song Wang

**Affiliations:** 1 Beijing Engineerin Research Center of Industrial Spectrum Imaging, School of Automation and Electrical Engineering, University of Science and Technology Beijing, Beijing 100083, Chinas20200534@xs.ustb.edu.cn (Y.L.); 2 Department of Traditional Chinese Medicine, Peking University People’s Hospital, Beijing 100044, China; 3 Department of Nephrology, Peking University Third Hospital, Beijing 100191, China; songwang30@163.com

**Keywords:** near-infrared image, blood vessel segmentation, noise reduction, image quality, mathematical morphology

## Abstract

The precise display of blood vessel information for doctors is crucial. This is not only true for facilitating intravenous injections, but also for the diagnosis and analysis of diseases. Currently, infrared cameras can be used to capture images of superficial blood vessels. However, their imaging quality always has the problems of noises, breaks, and uneven vascular information. In order to overcome these problems, this paper proposes an image segmentation algorithm based on the background subtraction and improved mathematical morphology. The algorithm regards the image as a superposition of blood vessels into the background, removes the noise by calculating the size of connected domains, achieves uniform blood vessel width, and smooths edges that reflect the actual blood vessel state. The algorithm is evaluated subjectively and objectively in this paper to provide a basis for vascular image quality assessment. Extensive experimental results demonstrate that the proposed method can effectively extract accurate and clear vascular information.

## 1. Introduction

Subcutaneous venipuncture is one of the common medical treatments encountered in daily life. As a category, it includes intravenous infusions and injections, and venous blood collection and transfusions [1]. In hospitals, most current methods of venipuncture require the use of a tourniquet fixed to a patient’s limb and then request the patient to clench a fist in order to make the blood vessel more prominent before puncturing it. Clearly, this method relies on the experience and proficiency of doctors or nurses, and there are many drawbacks to this method: one is that it is affected by skin pigmentation, vessel depth and thickness, and fat thickness [2]. Second, some patients fail to make a hand-clenched fist in some cases, resulting in the blood vessels not being prominently displayed. These conditions may lead to venipuncture failure, which not only increases the patient’s anxiety and pain but also brings psychological pressure to health care workers; sometimes, this also results in a risk of doctor-patient conflict. Therefore, the rapid and accurate acquisition of blood vessel location becomes a very important and urgent problem. The solution of this problem will help the evaluation of the quality of vessels, providing effective information to support nurses in their clinical care.

Many methods are currently proposed for blood vessel segmentation, and they are classified into two categories: the traditional methods and the machine learning methods. The traditional methods include the Gaussian function-based vessel segmentation, multiscale matched filtering, and threshold processing-based segmentation [3,4,5], etc. These methods usually need some preprocessing, such as the contrast limited adaptive histogram equalization (CLAHE) or Gabor filtering [6]. There is also a blood vessel feature extraction method which uses fuzzy theory to segment the veins [7]. The vessel tracking method, on the other hand, requires the initialization of a point on a vessel and then tracking of the localized vessel’s centerline by the monitoring of the image’s local information [8]. A vessel segmentation method based on the spatial feature point set of a rotating coordinate system clears off the error feature points, which has a good effect on peripheral blood vessels [9]. Some researchers have also proposed designing an active contour strategy coupled with Kalman filtering, which can be used to segment pulmonary vascular images [10].

The machine learning algorithm has two steps: the training step and image segmentation step. In the training step, the researchers use the segmented standard vascular images to train the classifiers according to certain learning methods. In the recognition segmentation phase, the unknown blood vessel images with certain features are fed into the trained classifier for segmentation. Support vector machine, convolutional neural networks, deep neural network [11,12,13], etc., are the representative methods. U-net is a typical deep learning network that can rely on data enhancement from a very small number of training images [14]. It has been used to segment human tissue images such as skeleton, muscle, and knee joints [15,16,17]. ResU-net focuses on solving problems such as tiny vessels at the end that are difficult to segment, and blurred edges caused by illumination [18]. R2U-net can enhance pixel correlation and increase the network depth effectively, having a good segmentation effect for lung CT images and fundus vascular images [19]. The LadderNet is essentially equivalent to the concatenation of two U-net networks [20]. The SMU-net proposes a saliency-guided morphology-aware U-net (SMU-net) for lesion segmentation, which tries to explore and exploit background-salient representations for assisting foreground segmentation [21].

Although many segmentation methods have been proposed, there are still many problems in their applications. The machine learning method has a high segmentation accuracy; however, its performance depends on the use of large amounts of data in the early stage to train the model. Due to the lack of unified calibration and tools, manual calibration by experts is still difficult. As a result, there is not enough standard dataset available for training currently. Therefore, it is not feasible to use the machine learning method to segment the near-infrared vascular images of forearm. Traditional segmentation methods do not need training, and their computation speeds are fast. However, traditional segmentation methods also have many shortcomings: first, because the edge of near-infrared vascular image is blurry, it is still hard to define the edge after enhancement, which requires higher criteria for the selection of threshold value in the traditional threshold segmentation. Second, the traditional segmentation is not effective at extracting some deep blood vessels and small blood vessels at the end. To address these problems of traditional segmentation algorithms, this paper proposes a vascular segmentation method based on background subtraction and improved mathematical morphology.

**Figure 1:**
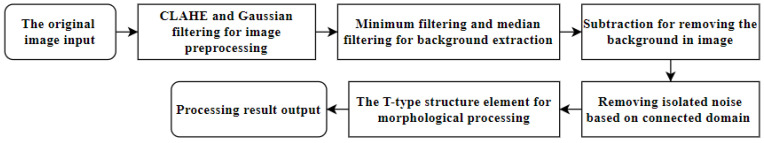
Proposed algorithm flow chart of near-infrared blood vessel image segmentation.

As shown in Figure 1, an improved vascular segmentation method using background subtraction and an improved mathematical morphology is proposed. Its computation can be roughly divided into the following steps: first, preprocessing, which improves the image brightness uniformity and prepares for image enhancement. Second, image background extraction, which uses the minimum filtering and median filtering to obtain image background information. Third, to obtain vascular information, the preprocessed image is used to subtract the extracted background information to obtain preliminary vascular information. Fourth, the method based on the connected domain is used to remove the isolated noises. Fifth, mathematical morphological processing is developed. This repairs some breakpoints in the preliminary results according to the state of blood vessel growth and the uniform blood vessel width is developed. The primary contributions of this paper include: (1) a vascular image segmentation method based on background subtraction is proposed, which ensures the accuracy of blood vessel assessments and simplifies the algorithm. (2) An improved mathematical morphology algorithm with a T-type structural element is proposed to extract information from blood vessels and smooth the edges of blood vessels.

## 2. Primary Computational Methods

### 2.1. Dataset Preprocessing and Enhancement

**Figure 2:**
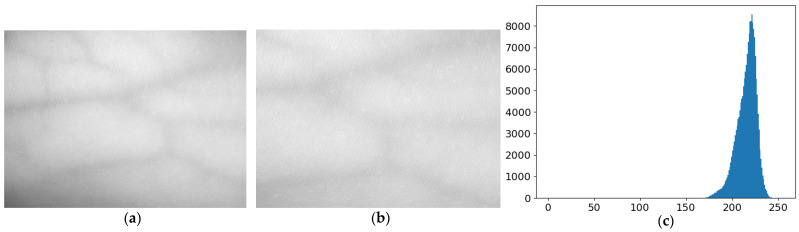
Image sample and its local vascular areas histogram. (**a**) An image sample from our dataset. (**b**) The local vascular areas image of (**a**). (**c**) The histogram of (**b**).

In previous work [22], we built a near-infrared image acquisition system and collected a forearm near-infrared vascular image dataset containing 360 images. Figure 2a shows an image sample taken from this dataset. It can be seen that the vascular image of the forearm is characterized by low contrast and uneven brightness distribution in imaging area, and that the finer blood vessels cannot be clearly observed under the light source irradiation. This is because the background of the human vascular region is complex, the difference between muscle and vascular region is not clear, and the imaging effect is easily affected by factors such as shooting angle, light source distribution, and other factors. Figure 2b presents its local vascular area of the image. It is not difficult to find that the edges of blood vessels in the image are fuzzy. However, it is difficult to observe the thinner blood vessels, and that there are a lot of noises. Figure 2c is the gray histogram of the local image Figure 2b. The gray distribution in the figure is concentrated, which indicates that the image is brighter overall and the contrast between foreground and background of blood vessels is low. In order to improve image contrast and enhance its details, this paper applies CLAHE to improve image contrast [23].

CLAHE is improved based on adaptive histogram equalization (AHE), which makes up for the noises brought by the AHE algorithm while enhancing image contrast [24]. The basic idea of CLAHE is to divide the image into a number of non-overlapping sub-regions and calculate the gray gradient value according to the statistics of gray level in each sub-region. When the local contrast of image is reduced or increased, CLAHE will automatically adjust, avoiding the problem of local excessive light or darkness. CLAHE’s specific steps are as follows.

First, CLAHE segments the original low-contrast image into M×N non-overlapping sub-regions and then calculates their local histograms. Second, CLAHE constrains the grayscale height of each local histogram and calculates the average number of pixels assigned to, the calculation method is shown in Equation (1):

(1) $$A_{\nu }=\frac{u_{X}u_{Y}}{N_{XY}}$$

where $N_{XY}$ denotes the number of gray levels in the corresponding subregion, and $u_{X},\;\mathrm{and}\;u_{Y}$ are the number of pixels in the *X* and *Y* directions of the subregion, respectively.

Third, CLAHE calculates the extreme values for which cropping is required, and calculate according to Equation (2):

(2) $$L_{C}=N_{CLip}A_{v}$$

where $N_{CLip}$ is the factor to limit the amplitude.

Fourth, let *S* mean the total number of pixels cropped, then the number of “cropped” pixels can be calculated as Equation (3):

(3) $$a_{v}=\frac{S}{N_{XY}}$$

Finally, the step size for assigning the remaining pixels is calculated as Equation (4):

(4) $$L=\frac{L_{G}}{S}$$

where *L* denotes the step size to be assigned to the remaining pixels and $L_{G}$ is the length range of grayscale.

Due to the imperfections in the formation, transmission, and recording of digital image signals, these factors can cause the contamination of digital image signals with a variety of noises. This can disrupt the observation of useful information in images, degrade image quality severely, and affect the enhancement and edge segmentation of blood vessel images. Therefore, this is an indispensable step to suppressing the noises of blood vessel images while trying to keep that the image quality detail features are not affected. In this paper, Gaussian filtering is chosen to suppress the noise just because it can effectively remove spot noises while preserving the edge information of an image [25]. Gaussian filtering is a linear filter that can smooth an image and make it more homogeneous. It operates by using a Gaussian function to perform a convolution operation. The two-dimensional Gaussian function is given by:

(5) $$G(x,y)=\frac{1}{2\pi \sigma^{2}}e^{-\frac{x^{2}+y^{2}}{2\sigma^{2}}}$$

where *x* and *y* denote the positions of the convolution kernel, respectively; additionally, σ is the standard deviation.

**Figure 3:**
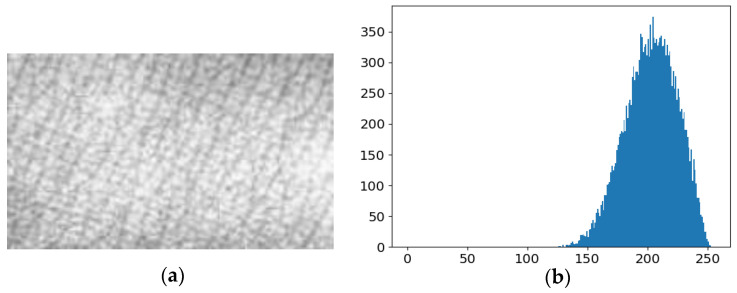
Local image of near-infrared blood vessel and its gray histogram. (**a**) Local image of near-infrared blood vessel. (**b**) The histogram of (**a**).

There are two reasons for using Gaussian filtering used in this paper. On the one hand, the Gaussian function can simulate the distribution of many phenomena in nature, such as sound, light, temperature [26], etc. The noise generated by the imaging sensor under many conditions belongs to Gaussian noise. On the other hand, the local image with relatively uniform brightness and non-vascular region in the near-infrared vascular image is captured and its gray histogram is calculated. As Figure 3b, from histogram, it can be found that its probability density function is similar to the density function of Gaussian noise distribution. Therefore, it can be concluded that the primary noise type in the near-infrared image is Gaussian noise. In this paper, the convolution kernel of 3×3 with σ=1.5 is used to convolve the image, and the noises in the image will be removed smoothly after Gaussian filtering.

### 2.2. Vascular Information Extraction and Image Background Subtraction

It is not difficult to observe that the grayscale value of the vascular region is lower than that of the non-vascular region, showing that the blood vessel is darker compared to the non-vascular region. According to this feature, the near-infrared vascular image can be regarded as a combination of background and vascular information. The image can be expressed as follows:

(6) I(x,y)=R(x,y)+G(x,y)

where I(x,y) denotes the near-infrared vessel image information, R(x,y) is the image information of vessel region, and G(x,y) indicates the background image information.

According to Equation (6), it is not difficult to derive that:

(7) $$R\left(x,y\right)=I\left(x,y\right)-G(x,y)$$

The function G(x,y) can be obtained as a background by applying minimum filtering and median filtering methods. Minimum filtering is a relatively conservative method of image processing. The processing principle of it is that the surrounding pixels and the center pixel value are first sorted. Then, the center pixel value is compared with the minimum pixel values. If the center pixel is smaller than the minimum value, the center pixel is replaced by the minimum value. In this paper, small circular regions with different gray values appear in the image after minimum filtering with 3×3 template. In the image, the difference of gray values on both sides of the inner and outer edges of blood vessels increased, and the number of different gray values decreased, which is helpful for clarifying the edge of blood vessels.

After the processing, the image background can be obtained by using the median filtering algorithm, which is a nonlinear signal processing technique based on the statistical theory of sorting, which can effectively suppress noises [27]. The basic principle of median filtering is to replace the value of a point in a digital image or digital sequence with the median of the values of points in a neighborhood of the center point, so that the surrounding pixel values are closed to the true value. Clearly, median filtering can blur the blood vessel image effectively. In this paper, the median filtering uses a two-dimensional sliding template of some structure to sort the pixels in a plate according to the size of pixel values, generating a monotonically increasing (or decreasing) tendency for two-dimensional data sequences. The expressions are:

(8) $$g\left(x,y\right)=Med\underset{\left(i,j\right)\in Sxy}{\left[f\left(i,j\right)\right]}$$

where *S_xy_* is the set of neighborhoods centered on (*x*, *y*) and Med is a function to take the middle value of the set.

### 2.3. Image Denoising and Morphological Processing

After subtracting the background operation, there are still many noise points and breaks. Additionally, if these isolated noise points are not removed, they will affect the subsequent link to repair the edges of blood vessels. Therefore, a connectivity domain-based element screening method is used in this algorithm to eliminate the isolated noise points [28]. The connected-domain-based element screening method refers to the calculation of the size of each connected domain in an image to determine whether it is a noise point or not. Since most blood vessels have elongated structures, the isolated noise points are generally round and irregular in shape, and their areas are much smaller than those of the connected blood vessel areas, a threshold can be set. If the area of the connected domain is smaller than a specific threshold, the connected domain can be considered as a noise region [29]. Its mathematical expression is:

(9) $$S_{n}=\left\{\begin{array}{cc} S_{n} & S⩾T\\ 0 & S<T \end{array}\right.$$

where $S_{n}$ represents the nth connected domain, S is the area of $S_{n}$, and T is the set threshold. In this algorithm, after a series of experiment tests, the T value can be set between 1500 and 1800 for our dataset, a value which can eliminate most of the noise points in blood vessel images.

After removing the isolated noise points, the edges of blood vessels may still have problems such as unevenness or even fractures. These problems do not correspond to the direction of normal blood vessels. To solve these problems, this paper develops an improved mathematical morphology algorithm. Mathematical morphology is a mathematical tool for image analysis based on morphological structural elements [30]. Its core idea is to use structures with certain morphologies to extract features such as the shape, structure, and spatial relationships of an image. When the template is continuously moved in an image, information about the interrelationships between various parts of the image can be collected to understand the image structure features. A novel mathematical morphology method of a T-type structural element is proposed in this paper. Using this method can allow researchers to simplify the image data, keep its basic shape features, and eliminate the skeleton of blood vessel.

The operations commonly used in mathematical morphology include dilation and erosion. In binary and grayscale images, each operation has its own unique characteristics. These basic operations can be further combined and derived to obtain various practical mathematical morphology algorithms. In dealing with the vascular edge problem, the dilation and erosion operations of morphological algorithm are used first. The dilation operation can expand the range of vessel edges and make them smoother, while the erosion operation can reduce the range of vessel edges and make them clearer. By properly combining these two operations, it can become more accurate and produce finer blood vessel images. In a binary image, the erosion operation is defined as:

(10) $$\left(f⊖e)(x,y)=min\{\begin{array}{c} f\left(x+a,y+b\right)-e\left(a,b\right)|\left(x+a\right),\\ \left(y+b\right)\in D_{f}\;and\;(a,b)\in D_{e} \end{array}\right\}$$

where f⊖e indicates that e is used to erode f,f represents the original image, and $D_{f}$ and $D_{e}$ are the definition domains of f and *e*, respectively; f(a,b) is outside the definition domain of  f, assuming it is +∞.

In the binary image, the dilation operation is defined as:

(11) $$\left(f⊕e)(x,y)=max\{\begin{array}{c} f\left(x-a,y-b\right)+e\left(a,b\right)|\left(x-a\right),\\ \left(y-b\right)\in D_{f}\;and\;(a,b)\in D_{e} \end{array}\right\}$$

where f⊕e indicates that e is used to dilate, f represents the original image, and $D_{f}$ and $D_{e}$ are the definition domains of f and *e*, respectively; additionally, f(a,b) is outside the definition domain of f, assuming it is −∞.

**Figure 4:**
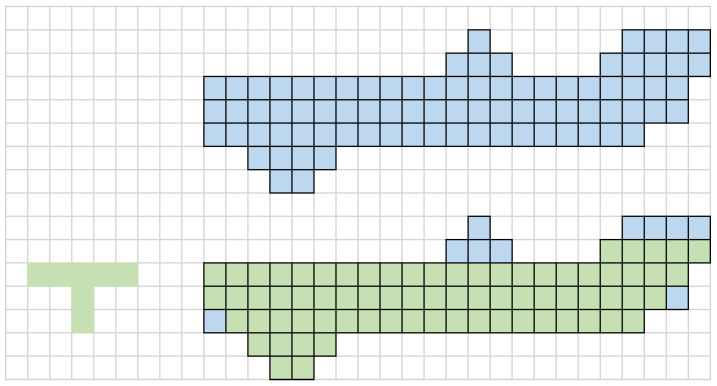
Proposed T-type structural element of mathematical morphology method.

The analysis of mathematical morphology theory shows that the selection of structural elements is crucial in the process of erosion and dilation, which directly determines the shape of processed image. The traditional structural elements are rectangles, circles, and crosses. However, in some cases, these structural elements do not fit well with the characteristics of an image and also do not perform well in the edge direction. Therefore, new structural elements need to be proposed to overcome this shortcoming. This paper proposes a novel T-type structural element. The advantage of this structural element is that it has high directionality, which can better process the line segmentations in different directions and smooth the protruding and depressed parts of image edges. As shown in Figure 4, the blue structure at the top is used to simulate a small segmentation of a blood vessel image, and the green structure at the bottom is the blood vessel structure corroded by a T-type structural element after background reduction and denoising. The vascular binary graph obtained initially has uneven edges, protrusions, or dents. The template of T-type structural element traverses all pixel points in image and retains the part that can completely overlap with this template, while the rest is corroded. As can be seen from the diagram, the T-type structural element effectively smooths some protruding parts, making the lines of the edge pixels smoother.

The algorithm in this paper is based on the T-type structural element, which is rotated by 90° in sequence and processed several times. In the algorithm, the following four structural elements are used to erode images.

(12) $$t_{1}=\left[\begin{array}{cccccc} 0 & 0 & 0 & 0 & 0 & 0\\ 0 & 0 & 0 & 0 & 0 & 0\\ 1 & 1 & 1 & 1 & 1 & 1\\ 1 & 1 & 1 & 1 & 1 & 1\\ 0 & 0 & 1 & 1 & 0 & 0\\ 0 & 0 & 1 & 1 & 0 & 0 \end{array}\right]$$

(13) $$t_{2}=\left[\begin{array}{cccccc} 0 & 0 & 1 & 1 & 0 & 0\\ 0 & 0 & 1 & 1 & 0 & 0\\ 0 & 0 & 1 & 1 & 1 & 1\\ 0 & 0 & 1 & 1 & 1 & 1\\ 0 & 0 & 1 & 1 & 0 & 0\\ 0 & 0 & 1 & 1 & 0 & 0 \end{array}\right]$$

(14) $$t_{3}=\left[\begin{array}{cccccc} 0 & 0 & 1 & 1 & 0 & 0\\ 0 & 0 & 1 & 1 & 0 & 0\\ 1 & 1 & 1 & 1 & 1 & 1\\ 1 & 1 & 1 & 1 & 1 & 1\\ 0 & 0 & 0 & 0 & 0 & 0\\ 0 & 0 & 0 & 0 & 0 & 0 \end{array}\right]$$

(15) $$t_{4}=\left[\begin{array}{cccccc} 0 & 0 & 1 & 1 & 0 & 0\\ 0 & 0 & 1 & 1 & 0 & 0\\ 1 & 1 & 1 & 1 & 0 & 0\\ 1 & 1 & 1 & 1 & 0 & 0\\ 0 & 0 & 1 & 1 & 0 & 0\\ 0 & 0 & 1 & 1 & 0 & 0 \end{array}\right]$$

According to the definition of dilation and erosion, this paper defines $f_{n}$ as the result of the *n*th processing. First, the $t_{1}$ structural element is used for erosion to remove small noise and fine lines on the edges; then, the circular template is also used to fill in some dotted lines and holes. The result of the first processing is shown below.

(16) $$f_{1}=\left(f⊖t_{1}\right)⊕b$$

where f is the original image and *b* represents the circular structural element.

For the second step, $t_{2}$ is used for corrosion and b is still used for dilation. The result of the second operation can be expressed as follows.

(17) $$f_{2}=(((f⊖t_{1})⊕b)⊖t_{2})⊕b)$$

Next, $t_{3}$ and $t_{4}$ are used for the same operation, and finally the processing result can be estimated by (13).

(18) $$f_{4}=\left(f_{3}⊖t_{4}\right)⊕b$$

Four calculations are completed in this step and the image is fully processed to obtain the desired shape. By using the T-type structural element and multiple rotations, this method can better deal with the problems of lines and voids in the binarized image and achieve a better denoising effect. This process can effectively eliminate horizontal and vertical line segments and noises.

## 3. Experiment Results and Evaluations

This section validates the noise reduction algorithm, segmentation algorithm, and morphological processing algorithm. The comparison tests randomly select forearm infrared blood vessel images from our dataset and crop each image into a region of interest (ROI) with a size of 548 × 335. Python programming is used for simulation experiments on PC Intel(R) Core(TM) i5-1035G1 CPU @ 1.00 GH, 8 GB RAM, NVIDIA GeForce MX 250).

### 3.1. Evaluation of Image Enhancement Algorithm

**Figure 5:**
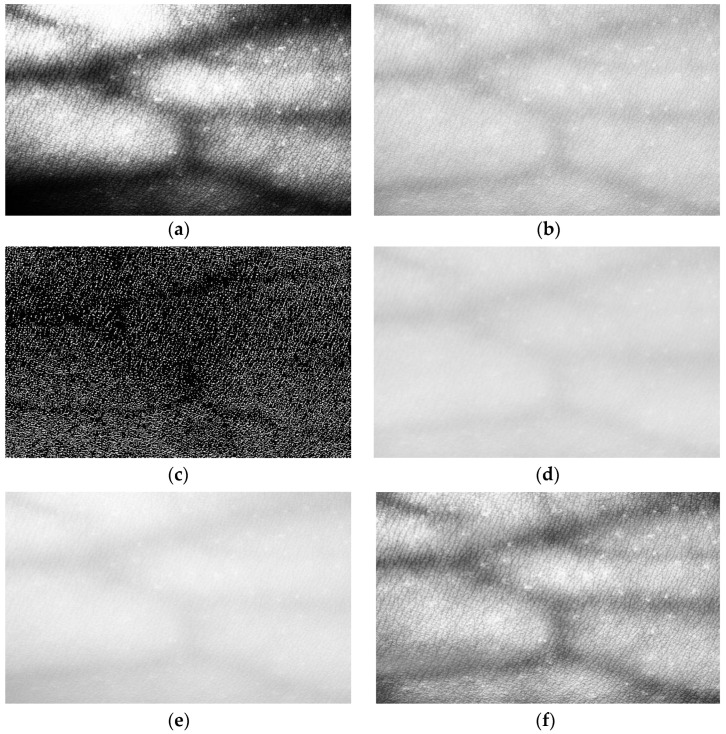
Experiment results of enhancement algorithms. (**a**) The result of HE. (**b**) The result of AHE. (**c**) The result of SSR method. (**d**) The result of BCMDBR. (**e**) The result of s homomorphic filtering. (**f**) The result of CLAHE.

To evaluate the enhancement effect further, five algorithms, i.e., CLAHE, histogram equalization (HE) algorithm [31], AHE algorithm [31], single-scale Retinex (SSR) algorithm [32], brightness compensation method for different brightness regions in the image (BCMDBR), and homomorphic filtering [33] are compared in this section. The results are shown in Figure 5. It can be observed from the results that, although the HE algorithm can improve image contrast, it can produce situations of local over dark or over bright images, and the effects of detail retention are not good. Although the above problems do not occur in AHE, noise is reflected and contrast enhancement is not as good as CLAHE. The SSR algorithm and BCMDBR appear to be over dark or over bright, and no vascular information can be observed. The homomorphic filtering algorithm is not effective for vascular edge processing, and its contrast is still not clear enough. Figure 5f shows the experimental results of the CLAHE. The forearm blood vessel image in ROI is investigated. The result shows that the CLAHE algorithm overcomes the problems of local overexposure and loss of details and can achieve better contrast enhancement while keeping the blood vessel images clearer.

### 3.2. Evaluation of Noise Reduction Algorithm

**Figure 6:**
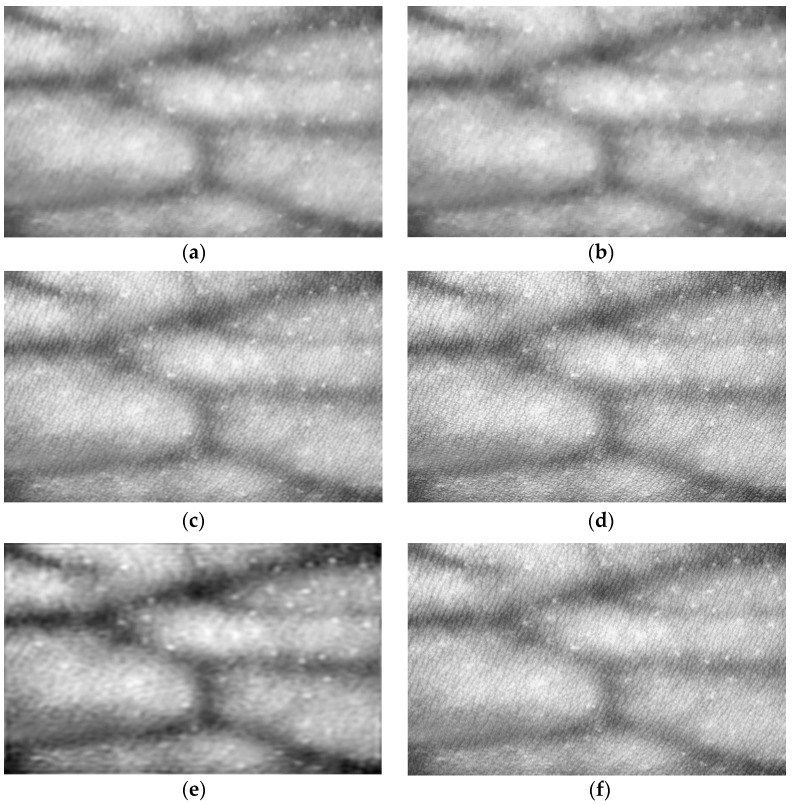
Experiment results of image noise reduction. (**a**) The result of bilateral filtering. (**b**) The result of median filtering. (**c**) The result of mean filtering. (**d**) The result of non-local-mean filtering. (**e**) The result of bandpass filtering. (**f**) The result of Gaussian filtering.

To compare the effect of noise reduction algorithms, the other traditional noise reduction methods like bilateral filtering [34], median filter algorithm [35], mean filter algorithm [36], non-local-mean filtering [37], and bandpass filtering are applied to the same noisy image for performance evaluation, respectively. The corresponding results are shown in Figure 6. From Figure 6, it can be observed that the bilateral filtering and mean filtering retain the edge information of images, but that the effects of noise removal are not as good as the effects of Gaussian filtering. The processing results of median filtering and non-local means denoising will cause the image to become blurred, and the bandpass filtering cannot remove the noise in the image well.

Since the image denoising evaluation is closely related to human vision and subjective perception, and human eye still has a certain deficiency in terms of detail discrimination, this paper introduces three metrics in order to further test the processing effect, i.e., the energy [38], Brenner [39], and visual information fidelity (VIF) [40]. This is carried out to evaluate image quality after denoising. The corresponding scores are shown in Table 1. Energy is an evaluation index used to measure the brightness value distribution, contrast, and other characteristics of an image. Generally, the higher the energy of an image is, the wider the distribution of luminance values and the stronger the contrast will be.

(19) $${D(f)=\Sigma }_{y}\Sigma_{x}(|f(x+1,y)-f(x,y){|}^{2}+|f(x,y+1)-f(x,y){|}^{2})$$

Brenner is an evaluation metric based on image gradient information. This method is usually used to measure image sharpness. The Brenner metric is sensitive to grayscale changes and can effectively distinguish the sharpness of various parts of an image.

(20) $${D(f)=\Sigma }_{y}\Sigma_{x}|f(x+2,y)-f(x,y){|}^{2}$$

VIF is an objective metric used to evaluate the quality of an image or video. It is based on the human visual system’s perception of information, and it also can quantify degrees of distortion. It takes into account several factors, including spatial frequency, brightness, or color, in order to more accurately simulate variation in perceived quality by the human eye. In the calculation of VIF function, multi-scale decomposition is firstly carried out, and then the information quantity is modeled and calculated at each scale. Finally, the VIF value is formed by combination. Higher VIF scores indicate better denoising effects. As shown in Table 1, the Gaussian filtering used in this paper has a good noise reduction effect on our dataset.

### 3.3. Evaluation of Image Segmentation Algorithm

**Figure 7:**
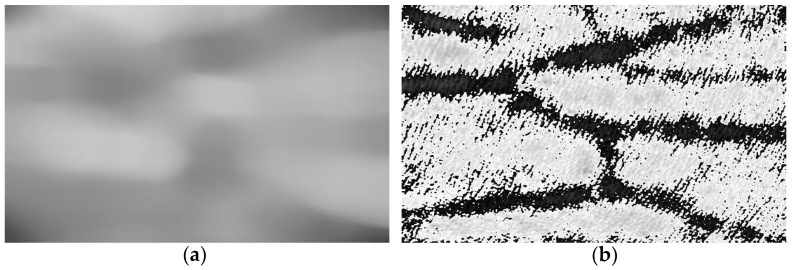
Processing result of background subtraction. (**a**) The background image extracted from the vascular image. (**b**) The extracted vascular image result.

Figure 7a shows the background of the near-infrared image obtained by minimum filtering processed by 3×3 template and the median filtering. The background image obtained can be considered as the luminance information without blood vessel information. By using Formula (7), the image information of the blood vessel region can be obtained without background information, as shown in Figure 7b. From the results, it can be observed that the background of near-infrared blood vessel image can be well extracted. Although there are some noises in the blood vessel information obtained by eliminating the background, the blood vessel information is well extracted without missing segmentation or wrong segmentation, and there are not many breakpoints in an image.

**Figure 8:**
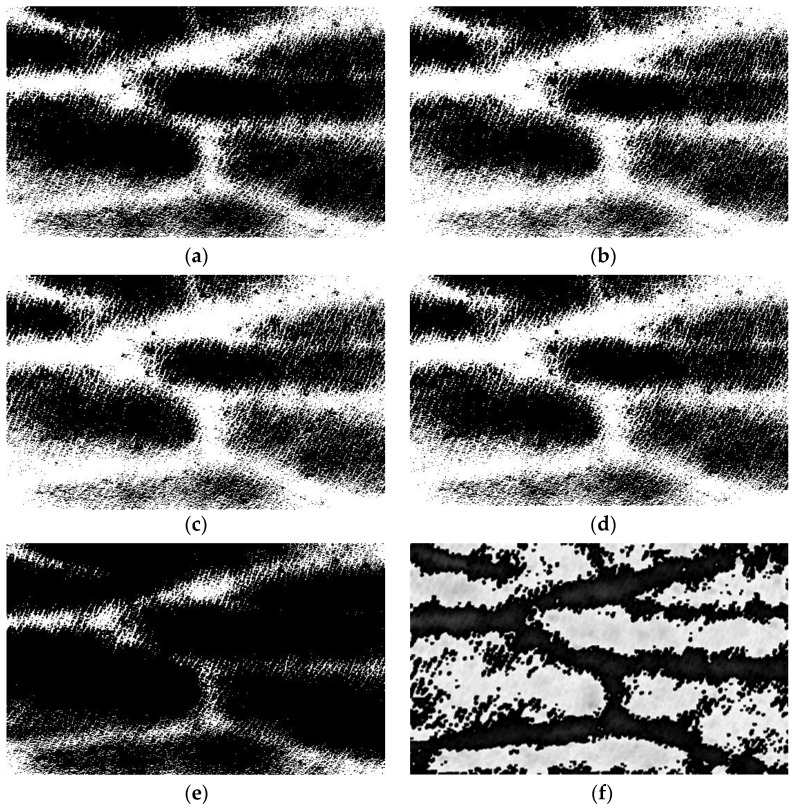
Experiment results of image segmentation. (**a**) The result of the global threshold-based segmentation. (**b**) The result of the region-growth-based thresholding method. (**c**) The result of the entropy thresholding method. (**d**) The result of the OTSU algorithm. (**e**) The result shows the histogram-based segmentation. (**f**) The result of our method.

To compare the experiment of segmentation algorithm performance, the global thresholding-based segmentation algorithm [41], region-growth-based thresholding method [42], entropy thresholding process [43], OTSU algorithm [44], and histogram-based segmentation algorithm [45] are applied to the same dataset to assess their processing effects, respectively. The results are shown in Figure 8. It can be observed that, although the segmentation algorithms based on global threshold, OTSU algorithm, and entropy threshold processing can segment the blood vessels, they cannot process the small-blood-vessel information well, and more details are lost. The segmentation effects of the threshold method assessed based on region growth, alongside the algorithms investigated based on histograms, are not good, respectively. Even for the primary blood vessels, the blood vessels cannot be clearly identified. In contrast, the algorithm in this paper does not omit details when considering the primary blood vessels, and its use can segment a relatively clear and complete vascular framework. This is because the traditional segmentation algorithms are based on the threshold value, which can be considered as the vascular feature. The segmentation algorithm based on the global threshold is to choose the manual threshold, and it needs to be adjusted continuously to obtain a more appropriate value, meaning that the algorithm is not robust. Based on the histogram technique and other algorithms, the appropriate threshold value can be obtained through calculation, but the easy threshold value will be affected by many factors in the calculation process, such as brightness, noise, etc. There are high requirements for using the pre-processing algorithm, and its use is not suitable for processing near infrared vascular images. However, our image reduction algorithm does not use threshold value and is less affected by brightness and other factors, and as a result it has a good effect.

Some objective quantitative analyses are also performed. Three indexes are considered in this paper, i.e., Dice, accuracy (Acc), and sensitivity (Sen) [46,47]. The Dice coefficient is a function to evaluate similarity. This is usually used to calculate the similarity or overlap of two samples. Its computation method is defined in (11). Acc indicates the proportion of correctly segmented pixel points in segmentation result to the total number of pixel points in the whole image. Its formula can be calculated by (12). Sen means the proportion of correct vessel points in the segmentation results to the total number of vessel points in the standard segmentation result [47]. Its calculation method is illustrated in (13).

(21) $$Dice=2\frac{V_{seg}\cap V_{gt}}{V_{seg\;}{\cup \;V}_{gt}}$$

(22) Acc=(TP+TN)/(TP+TN+FP+FN)

(23) Sen=TP/(TP+FN)

where $V_{seg}$ denotes the segmentation result, $V_{gt}$ means the expert segmentation result; $V_{seg}\cap V_{gt}$ represents the intersection of $V_{seg}$ and $V_{gt}$, $V_{seg},and\;{\cup V}_{gt}$ represents the union of $V_{seg}$ and $V_{gt}$. True positive (TP) shows the correctly segmented vessel points; false positive (FP) indicates the incorrectly segmented vessel points; true negative (TN) indicates the correctly segmented background points; and false negative (FN) means the incorrectly segmented background points [47].

Table 2 presents the corresponding results. Table 2 shows that, although the Acc score of our method is not as good as those of other algorithms, the reason for the low Acc in the segmentation evaluation results in this paper is the class imbalance in the vascular images. Vascular segmentation is essentially a classification problem. In near-infrared vascular images, the area of non-vascular regions is much larger than the area of vascular regions, causing class imbalance and low Acc. In contrast, Dice and Sen focus more on the degree of overlap between prediction and ground truth. As a result, Dice and Sen are higher, which also reflects that the segmentation effects in this paper are closer to the manual segmentation results of experts.

### 3.4. Evaluation of Morphological Algorithm

**Figure 9:**
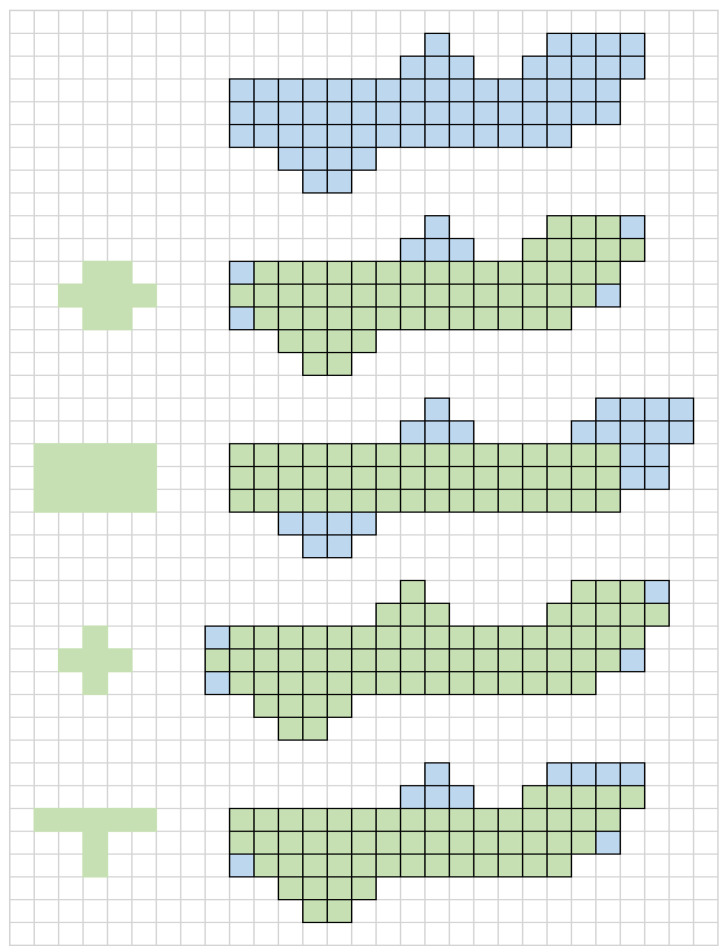
Processing effect examples of different shape structural elements.

This paper assesses the morphological processing algorithms. The traditional corroded and inflated structural elements, whether elliptical, rectangular, or cross-type, are compared with T-type structural element to obtain the same dataset of binarized images. In order to facilitate the understanding of the advantages of T-type structural elements, this paper firstly draws pictures to demonstrate the working process of ellipse, rectangular, cross-type and T-type structures. As can be seen from Figure 9, although the ellipse structure cannot handle the upper and lower edge structures, the gentle convex part of them cannot be processed well. The rectangular structure can remove the convex part of edge better, but the angles of shape details are lost. The cross-type structure has little effect on the sharp protrusions. The T-type structural element can retain as much shape information as possible, and it can also “smooth” the convex part of the upper and lower edges.

**Figure 10:**
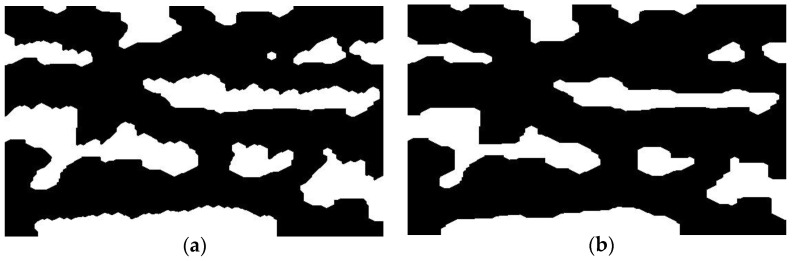

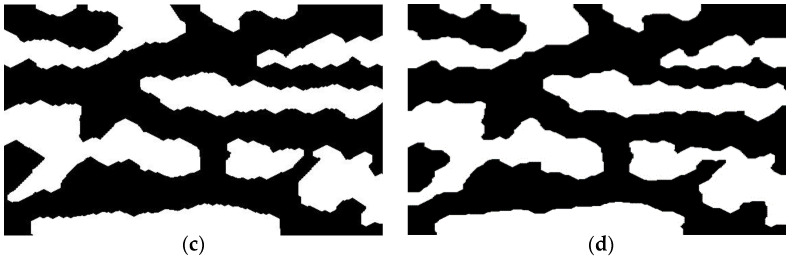
Experiment results of morphologic processing. (**a**) The result of elliptic structural elements. (**b**) The result of rectangular structural elements. (**c**) The result of cross-type structural elements. (**d**) The result of T-type structural elements.

Figure 10 shows the corresponding processing results of the blood vessel images using the T-type, elliptic, rectangular and cross-type structural elements. It can be seen from Figure 10 that the morphologic processing algorithm based on T-type structural element can remove the noise and holes better and extract the target region more accurately. Compared with the traditional ellipse type and cross-type, the T-type structural element smooths the upper and lower edges better. Although the rectangular structural element has a better smoothing effect, it displays more distortions compared with the real blood vessel region. Therefore, a T-type structural element is considered better able to adapt to the characteristics of blood vessel image, which can improve the effect of image processing to a certain extent.

## 4. Discussion

In this research, a method based on background subtraction and improved morphology is proposed to design an effective preprocessing and segmentation method for forearm near-infrared blood vessel images. Not only the gray characteristics, but also the morphological features, are considered when designing this algorithm. The target vascular region is accurately processed according to the relevant morphological characteristics in order to ensure segmentation accuracy. The algorithm has a better anti-noise performance than the traditional algorithm. The proposed algorithm aims to improve the extracted blood vessel information by applying several steps: firstly, the original image is preprocessed with CLAHE to achieve a highlighted vascular part. Then, Gaussian filtering is applied to reduce the noise in the image. Next, the background image is obtained by blurring the image with minimum filtering and median filtering. It is then subtracted from the enhanced image to obtain a preliminary binary image of a blood vessel. In the segmentation step, the isolated noises are removed using the element screening method based on the connected domain. Then, the image is dilated and eroded with T-type structural elements to render uniform the width of blood vessels and smooth edges. Through these steps, the vascular region in the forearm near-infrared venous vessel image can be effectively extracted with better performance and accuracy than other existing methods.

**Figure 11:**
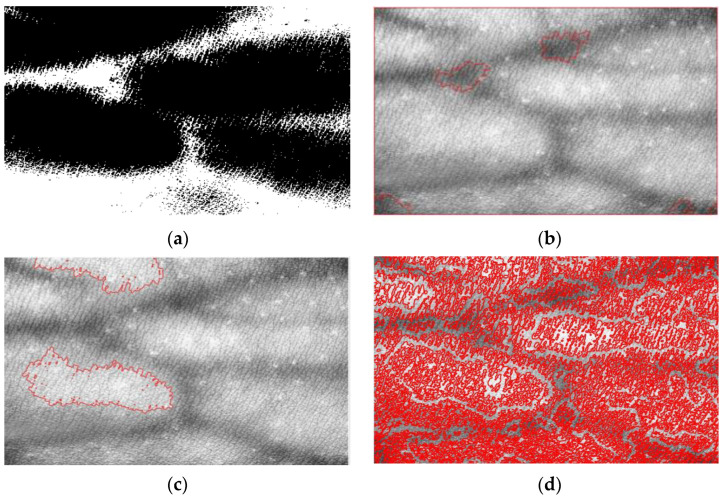
Comparison results of other segmentation algorithms. (**a**) The result of K-means. (**b**) The result of watershed algorithm. (**c**) The result of active contour method. (**d**) The result of LBF.

At present, there are limited datasets for near-infrared vascular images, and manual segmentation of vascular images cost lots of manpower; therefore, it is difficult to use supervised learning method to segment images. This paper also tries other techniques to segment the blood vessel image. For example, the K-means clustering algorithm [48], watershed algorithm [49], active contour method [50], and the method of implicit active contours, driven by local binary fitting energy (LBF) [51], are all considered. According to the results which are shown in Figure 11, the first three algorithms cannot segment the near-infrared vascular images with poor results, and cannot extract the blood vessel skeleton with more details lost. The segmentation result of LBF the method exhibit a serious over-segmentation phenomenon, and the blood vessel information cannot be extracted in the image. The primary reasons for these phenomena are as follows: the K-means segmentation is based on gray level segmentation in essence, which has high requirements on illumination changes, initial clustering center selection, and other factors. Additionally, the watershed segmentation easily produces segmentation deviation and has poor effects on images with low contrast. The algorithm model of active contour is dependent on parameters, and suitable parameters should be found for different images to achieve good segmentation effect. Additionally, the LBF method has high requirements for application to an the image, and when image noises are strong, its segmentation effects are not accurate.

This study is of great significance for the field of medical image processing, which can improve the quality and readability of near-infrared vascular images and provide more accurate and effective information for clinical diagnosis and treatment. In addition, this study provides a new non-invasive detection method for the field of cardiovascular disease. It can be used to assist nurses in the evaluation of vascular quality and help doctors in the detection, localization and intervention to treat venous thrombosis, which is helpful in efforts to improve the quality of life and safety of patients. However, this study also has some limitations. These must be addressed in the future. For example, this study only verifies the algorithm on forearm near-infrared vascular images without considering other parts or other types of vascular images. The repair effect of this paper needs to be improved in relation to some fractures. Based on these limitations, future research can be carried out in the following aspects: first, the cooperation with hospitals to collect more data will be established for expanding the scope of the experiment, which will test the effect of the algorithm on other sites or other types of vessel images. Second, the ability of the algorithm to deal with more details of the blood vessels will be improved, and efforts will be constantly made to design a corresponding solution or improvement strategy will.

## 5. Conclusions

In this paper, background subtraction and improved mathematical morphology technique are used to extract blood vessel information for the task of forearm infrared blood vessel segmentation. Initially, image enhancement and spatial domain filtering are used to enhance near-infrared vascular images and highlight the target vascular region. Secondly, the fuzzy method is used to obtain the image background, and then the idea of removing the background from the image is used to obtain the blood vessel information. Finally, the element screening method based on the basic operation of connecting domain and enhanced morphology is used to remove noise and smooth edges in order to ensure the uniformity of the width of the target blood vessel region. Compared with traditional vascular detection technology, the method proposed in this work is simpler, more intuitive, efficient and accurate, and can provide valuable information and support for non-invasive medical diagnosis and treatment. In addition, it has a wide range of possible applications in medical image processing and is likely to make greater contributions to the field of medical diagnosis in the future.

## Figures and Tables

**Table 1:** Evaluation results of different noise reduction algorithms.

Name of Filtering Algorithm	Energy	Brenner	VIF
Bilateral filtering	0.111113	0.149405	0.0229357
Median filtering	0.142327	0.185371	0.0171459
Mean filtering	0.542328	0.717005	0.228890
Non-local means denoising	0.166525	0.200364	0.192109
Bandpass filtering	0	0	6.829285 × 10^−5^
Gaussian filtering (Our method)	0.579129	0.788554	0.314532

**Table 2:** Evaluation results of different segmentation algorithms.

Name of Filtering Algorithm	Dice	Acc	Sen
Threshold segmentation method based on region growth	0.66151	0.570808	0.668102
Threshold method based on regional growth	0.670982	0.489470	0.811824
Entropy threshold method	0.662919	0.456471	0.8487629
OTSU threshold segmentation algorithm	0.670982	0.489471	0.811824
Histogram-based technique for threshold segmentation	0.457068	0.657206	0.319836
The algorithm in this paper	0.697933	0.443855	0.938922

## Data Availability

The data presented in this study are available on request from the corresponding author Haoting Liu.
